# Transcranial direct current stimulation over the temporal-parietal junction yields no lexical-semantic effects in logopenic primary progressive aphasia: a double-blind sham-controlled study

**DOI:** 10.1016/j.nicl.2025.103798

**Published:** 2025-05-05

**Authors:** Marc Teichmann, Clara Sanches, Angelina Bourbon, Dennis Q. Truong, Marom Bikson, Antoni Valero-Cabré

**Affiliations:** a Pitié Salpêtrière University Hospital, Department of Neurology, National Reference Center for ‘Rare or Early Onset Dementias’, AP-HP Paris, France; b Institut du Cerveau et de la Moelle Epinière, ICM-UMR INSERM-CNRS-SU 1127, Frontlab team, Paris, France; c Institut du Cerveau et de la Moelle Epinière, UMR INSERM-CNRS-SU 1127, Groupe de Dynamiques Cérébrales, Plasticité et Rééducation, ICM, CNRS UMR 7225, Paris, France; d University of Geneva, Department of Psychology and Educational Sciences, Geneva, Switzerland; e Laboratoire de Phonétique et Phonologie, UMR7018, CNRS, University Sorbanne Nouvelle. Paris, France; f Neural Engineering Laboratory, Department of Biomedical Engineering, The City College of City University of New York, New York, NY, USA; g Laboratory for Cerebral Dynamics Plasticity and Rehabilitation, Boston University School of Medicine, Boston, MA, USA; h Cognitive Neuroscience and Information Technology Research Program, Open University of Catalonia (UOC), Barcelona, Spain

**Keywords:** tDCS, Logopenic primary progressive aphasia, Language

## Abstract

Transcranial direct current stimulation (tDCS) has generated some promising outcomes in primary progressive aphasia (PPA). The logopenic variant (lv-PPA), one of the most frequent PPA phenotypes, erodes the temporal-parietal junction (TPJ) generating impaired lexical processing, rapidly extending to semantic deficits. Positive tDCS effects have been reported in several small-cohort studies but there is need for rigorous sham-controlled double-blind investigations to substantiate, or not, beneficial effects.

We used a sham-controlled double-blind counter-balanced crossover design with 12 clinically and imaging-characterized lv-PPA patients applying, according to the principle of interhemispheric rivalry, anodal and cathodal tDCS over the left and right TPJ, respectively, as compared to sham. A letter fluency (lexical access), a picture-naming (lexical/semantic access), and a semantic-matching task (semantic access) were applied before and after tDCS. Computational modeling was used to characterize predicted cortical tDCS current distribution.

Comparisons of post/pre-tDCS results did not show language improvement in any task. Finite element models showed impact for both tDCS modalities on the TPJ, but with lower radial field-strength when atrophy was implemented in the model. Correlation analyses on individual data, uncorrected for multiples comparisons, suggested that lesser aphasia severity and shorter disease duration are associated with more efficient tDCS effects.

Our results showing the absence of significant tDCS outcomes in lv-PPA mitigate previous reports of positive tDCS effects with similar or smaller patient sample sizes, and they demonstrate the need for exploring factors influencing stimulation effects. Findings from computational modelling combined with our uncorrected correlation results suggest that tDCS use might be most appropriate in PPA patients having slight atrophy and aphasia severity. Future studies on larger patient populations are required for robust proof-of-concept regarding therapy use of tDCS in PPA.

## Highlights

• This sham-controlled double-blind tDCS study revealed no language effects in lv-PPA.
• Computational modelling (CM) implementing atrophy showed reduced radial field-strength.
• CM and correlations with individual data suggest that tDCS might be most efficient in lv-PPA with mild atrophy/aphasia.
• Best tDCS targets in PPA might be non-atrophied regions of the brain-language network.

## 1 Introduction

Primary progressive aphasia (PPA) designates a group of neurodegenerative disorders affecting language processing (Mesulam et al., 2014). Among the three main phenotypes (semantic, nonfluent/agrammatic, logopenic) the logopenic variant (lv-PPA) causes damage to the processing of lexical information and to verbal short-time memory. Both are implemented by the temporal-parietal junction (TPJ) of the dominant hemisphere and its erosion in lv-PPA leads to impairment of word finding and to breakdown of sentence repetition/comprehension (Gorno-Tempini et al., 2011). Lv-PPA is a fast evolving disease (Rogalski et al., 2011, Leyton et al., 2013) often leading to semantic impairments after 3–4 years of disease evolution (Leyton et al., 2013, Teichmann et al., 2013, Patel et al., 2021). Accordingly, several investigations have shown that lv-PPA patients demonstrate, in addition to lexical disorders, semantic deficits reflected by semantic paraphasias, diminished semantic priming or reduced performance in semantic matching (Teichmann et al., 2013, Wicklund et al., 2014, Migliaccio et al., 2016, Sanches et al., 2018). There is no pharmacological treatment for PPA, and language training has shown modest outcomes. A promising treatment approach is non-invasive brain stimulation (NIBS) that allows for directly interacting with neural networks. In the field of aphasia, NIBS such as transcranial magnetic stimulation (TMS) or direct current stimulation (tDCS), has initially been applied to post-stroke aphasia. Despite heterogeneous outcomes, studies have provided three main findings. First, NIBS over cortical language regions can generate positive effects, whereas targeting non-language specific regions does not (Monti et al., 2008). Second, most beneficial effects are reported after anodal (‘excitatory’) tDCS or high-frequency TMS over damaged left-sided language regions, or after cathodal (‘inhibitory’) tDCS or low-frequency TMS over homotopic regions of the non-dominant hemisphere (Hamilton et al., 2011). These latter effects are thought to be related to the principle of interhemispheric rivalry (Ferbert et al., 1992). Third, applying NIBS over the TPJ in post-stroke aphasia has yielded positive effects in tasks involving lexical processing such as picture-naming (Fiori et al., 2011). A second line of evidence for potential therapeutic utility derives from NIBS studies reporting beneficial effects in PPA variants including lv-PPA (Cotelli et al., 2014, Gervits et al., 2016, Roncero et al., 2017, Ficek et al., 2018, Tsapkini et al., 2014, Tsapkini et al., 2018). However, results are mitigated by methodological issues, especially the absence of double-blind and/or sham-controlled designs in most of prior investigations (Sanches et al., 2021 for a review). Furthermore, most NIBS studies in lv-PPA did not target the TPJ but rather non-language specific prefrontal regions.

With the aim of checking for NIBS effects we conducted a tDCS investigation in lv-PPA using a double-blind, sham-controlled, randomized and counter-balanced cross-over design, in which we applied anodal and cathodal tDCS over the TPJ of the dominant/left and non-dominant/right hemisphere, respectively. Both stimulation modalities were compared to sham tDCS. Significant results sought to provide proof-of-concept for future investigations with multiday tDCS regimes in large patient cohorts for potential therapeutic validation of tDCS use. On the other side, absence of tDCS effects would allow for delineating factors influencing NIBS effects in lv-PPA, and more generally in PPA.

## 2 Methods

### 2.1 Participants

Twelve patients satisfying the international diagnostic criteria for lv-PPA (Gorno-Tempini et al., 2011) were recruited at the Reference Center for “Rare or Early Onset Dementias” at the Pitié-Salpêtrière University Hospital, Paris, France. At time of diagnosis all patients had largely predominant language impairments, presenting the core features of lv-PPA: word-finding difficulties in spontaneous speech and picture-naming, and sentence repetition impairments, in the absence of agrammatism, semantic or motor speech disorders. Diagnoses were also imaging supported showing isolated/predominant damage to the left TPJ on FDG-PET, sometimes also visible on generally less sensitive MRI explorations. Patients were included in the study approximately 4 years after symptom onset, and around 2 years after the initial diagnosis. Exclusion criteria were (i) psychiatric/neurologic diseases other than lv-PPA, (ii) contra-indications for MRI or tDCS (intracranial ferromagnetic devices, scalp/skull lesions, epilepsy), (iii) major depression (MADRS > 20 [Montgomery Asberg Depression Rating Scale] (Montgomery and Asberg, 1979), or major cognitive disorders (MMSE < 15 [Mini-Mental State Examination] (Folstein et al., 1975), FAB < 10 [Frontal Assessment Battery] (Dubois et al., 2000). Fifteen healthy controls, with similar characteristics as patients for handedness (all right-handed), gender, age and years of education (Chi-square for gender: p > 0.05; Mann-Whitney for age and years of education: both p-values > 0.05) were included to determine normative performance levels in the experimental language tasks. All participants were native French speakers. Demographic data are summarized in Table 1. Informed consent has been obtained from all participants and privacy rights have been fully respected. The study conformed to the Declaration of Helsinki, to French regulations, and was approved by the ethic committee (ID RCB/IRB N°:2013-A00734-41).

**Table 1:** Demographic data of lv-PPA patients and healthy controls (mean scores ± standard deviations).

	**lv-PPA patients**	**healthy controls**
**Number of participants**	12	15
**Sex (women, men)**	6 W/6M	8 W/7M
**Age (years)**	70.5 ± 2.6	64.1 ± 7.4
**Handedness (R/L)**	12/0	15/0
**Years of education (years)**	14.1 ± 1.0	14.9 ± 2.7
**Symptom duration (years)**	4.3 ± 0.7	–

### 2.2 Study design

The design was similar to that used in previous studies of our group exploring semantic PPA (Teichmann et al., 2016), progressive supranuclear palsy (Valero-Cabré et al., 2019; and frontotemporal dementia (Sanches et al., 2022). We applied a double-blind sham-controlled crossover design, with each patient undergoing three tDCS sessions: anodal and cathodal tDCS over the left and right TPJ, respectively, and sham stimulation over the left TPJ. We used tDCS rather than TMS because it allows for a wider range of cortical action (Torres et al., 2013), and because tDCS material can be easily used for clinical applications (Elder and Taylor, 2014). Stimulation was preceded, and immediately followed, by three language tasks to evaluate potential stimulation effects. The order of the three stimulation sessions was counterbalanced across the 12 patients to avoid order biases (6 permutations × 2), and the sessions were one week apart to prevent any carry-over effects. The lack of intense/lasting scalp sensations made patients unaware of the tDCS condition. To guarantee a double-blind procedure, two different researchers supervised the application of tDCS and of the language tasks.

### 2.3 MRI-voxel-based morphometry

MRI imaging and analyses were conducted to support lv-PPA diagnostic, to localize cortical thinning in patients as compared to controls, and to identify TPJ coordinates for the placement of tDCS electrodes. T1-weighted MRI has been performed for the 12 patients and for 11 healthy controls, using a 3 T scanner (VERIO system, SIEMENS, Germany) with a 32-channel head coil including anatomical 3D T1-weighted MPRAGE images (magnetization prepared rapid acquisition gradient echo; TR = 2.3 s; TE = 4.18 ms; flip angle = 9°; TI = 900 ms; voxel size = 1 × 1 × 1 mm^3^; 176 slices). Image processing and statistical analyzes were performed using the software SPM 12 (Wellcome Department of Cognitive Neurology, UK), running on Matlab 2016b (Mathworks, USA). Image preprocessing included the following steps: segmentation, spatial normalization, modulation and smoothing. T1 images were segmented and normalized to MNI space using DARTEL. The normalized and modulated images were then smoothed using a Gaussian kernel of 10 mm full-width-at-half-maximum (FWH). Voxel-based morphometry (VBM) was used to compare gray matter volumes between healthy controls and patients using a two-sample *t*-test design. Age, gender, and total intracranial volume were introduced as nuisance variables. We tested two statistical thresholds: p < 0.05 with family-wise error (FWE) correction for multiple comparisons, and p < 0.001, uncorrected. The uncorrected threshold was accepted given the relatively small sample size of the two groups.

### 2.4 Brain stimulation

MRI data allowed for the precise placement of electrodes ensuring the shortest/straight path to the cortical target, using an MRI-guided stereotaxic neuronavigation system (Brainsight, Rogue Research, Canada). Targets for anodal and cathodal tDCS were located in the left and right TPJ, corresponding to the Montreal Neurological Institute (MNI) coordinates [x = −64, y = −38, z = 6] and [x = 64, y = −38, z = 6], respectively, which was consistent with prior studies (Roiser et al., 2013). A return electrode was placed over the contralateral supraorbital region. The scalp location of active tDCS electrodes corresponded to ∼CP5 (left TPJ) and ∼CP6 (right TPJ) according to the 10–20 EEG reference system, and the contralateral supraorbital return electrodes were placed on AF8 and AF7, respectively. During anodal and cathodal tDCS, current intensity was linearly increased for 30 s to a maximum of 1.59 mA, delivered through round sponge electrodes (5.65 cm diameter, 25 cm^2^ surface, NEuroelectrics [NE026a] SPONSTIM 25), ensuring a current density of 0.06 mA/cm^2^), similar to those applied in previous studies (Tsapkini et al., 2018, Teichmann et al., 2016, Valero-Cabré et al., 2019, Sanches et al., 2022). Current was kept at this intensity for 20 min before being ramped down for 30 s. For sham tDCS, the current was ramped up and down during 30 sec at the initial and final phase of the session to emulate potential scalp itching sensations that can characterize active stimulation. During the tDCS sessions patients performed a visuo-motor task consisting in pressing a response button every time a moving target touched the edge of a surrounding rectangle, ensuring that patients maintained vigilance. To assess tDCS tolerance, patients completed a tDCS adverse effects questionnaire (Brunoni et al., 2011).

### 2.5 Computational model simulations of current density distribution

An image-derived standardized head volume (ICBM-NY) (Huang et al., 2016) was used to produce Finite Element Method (FEM) models to determine electric field distributions throughout the head/brain. Models were generated for the two electrode montages used in the study. Electric field was projected onto the surface normal of the cortex to calculate inward (anodal) or outward (cathodal) fields. A model implementing cortical thinning of 1.5 mm in a region of a 5 cm diameter centered on the MNI target of the TPJ addressed the impact of TPJ atrophy on electric field current density levels impacting the left and right TPJ. Additional details on tDCS modeling procedures implementing identical parameters (electrode types, current intensity, conductivities, boundary conditions, field equations) are described in previous investigations of our group (Teichmann et al., 2016, Valero-Cabré et al., 2019, Sanches et al., 2022).

### 2.6 General cognitive/language assessment

Standard clinical tests contributed to lv-PPA diagnosis, and to the quantification of language/cognitive deficits. The general cognitive assessment included, among other neuropsychological tests, the MMSE (Folstein et al., 1975), the FAB (Dubois et al., 2000), and the MADRS (Montgomery and Asberg, 1979). The language assessment was composed of an evaluation of aphasia severity, a sentence-repetition test and a single-word comprehension test (Boston Diagnostic Aphasia Evaluation [BDAE] (Mazaux and Orgogozo, 1982), a picture naming test [D080] (Deloche and Hannequin, 1997), and a letter and category fluency test (Cardebat et al., 1990). Test scores are summarized in Table 2.

**Table 2:** General cognitive/language assessment (mean scores ± standard deviations).

**Standard Tests**	**lv-PPA patients**	**healthy controls**	**normative thresholds**
**MMSE** (/30)	21.8 ± 1.1	29.33 ± 0.72	≥27
**FAB** (/18)	11.6 ± 0.8	17.7 ± 0.1	≥16
**BDAE** – **aphasia severity scale** (/5)	3.75 ± 0.2	–	>4
**DO80** (/80)	63.2 ± 8.5	–	>75
**BDAE – sentence repetition** (/16)	10.2 ± 1.9	–	≥14
**Letter fluency** (P/2 min)	10 ± 3.4	–	≥15
**Category fluency** (fruits/2 min)	8.9 ± 4.2	–	≥15
**BDAE – single-word comprehension** (/72)	65.8 ± 7.6	–	≥68

MMSE = Mini-Mental State Examination, FAB = Frontal Assessment Battery, BDAE = Boston Diagnostic Aphasia Evaluation, DO80 = D080 picture naming test.

### 2.7 Experimental language tasks

We used three tasks taping access to lexical and semantic representations to measure whether tDCS leads to modulation/improvement of lexical-semantic capacities. The letter fluency task (LFT) mainly assessed access to lexical information whereas the picture naming task (PNT) probed for access to both lexical and semantic information. A semantic association/matching task (SAT) probed more specifically for access to semantic representations.

In the LFT participants were asked to produce orally as many words as possible beginning with a given letter within one minute (“C” or “P”). Letters were displayed on the computer screen and provided orally. Patients performed this task prior and following stimulation either with the letter “C” (Version 1) or “P” (Version 2) to avoid/reduce test/re-test effects. Words beginning with “C” or “P” are similar in terms of number of items and they have similar cumulative lexical frequencies in French (both Fs < 1) (New et al., 2004).

In the PNT participants were asked to name 40 pictures, issued from two picture databases (Snodgrass and Vanderwart, 1980, Bonin et al., 2003), as quickly and accurately as possible. Each picture was displayed on a computer screen for 8 sec, after which a lack of response was counted as an incorrect answer. Patients performed two different versions of the task prior and after stimulation to avoid test/re-test effects. For both versions of the task, the material was matched for visual complexity, lexical frequency and concept familiarity (all Fs < 1) (New et al., 2004). The number of correct answers and reaction times (RT) were recorded by the computer once the examiner clicked on the corresponding (correct/incorrect) button after responses were given by the participants.

In the SAT three words were presented on the computer screen: a test item (top), a semantically related target (bottom left or right), and an unrelated distractor (bottom right or left). In half of the stimuli the target was located at the left bottom side (distractor on the right), and in the other half at the right side (distractor on the left). Participants decided as accurately and as quickly as possible which of the two items at the bottom of the screen was related to the test item. There were two versions of the task to avoid test/re-test effects. Each version was composed of 26 stimuli. Test items, targets, and distractors of the two versions were matched for lexical frequency, number of letters and concept familiarity (all Fs < 1). Within each version targets and distractors were matched for lexical frequency, number of letters and concept familiarity (all Fs < 1) (New et al., 2004). Performance accuracy and RT were automatically recorded by the computer.

The order of the tests was blocked as follows: 1) LFT, 2) PNT, 3) SAT. Stimuli were presented on a laptop computer (HP EliteBook 8770w) with *E-Prime software*. The testing procedure required about 15 min, which is within the post-stimulation period covered by offline effects after 20 min tDCS (Nitsche and Paulus, 2001). Testing healthy controls allowed for determining normative performance/RT levels.

## 3 Statistical analyses

To characterize language impairments in patients, performances and RT at baseline (pre-stimulation sessions) were compared with those of the 15 controls using a non-parametric Wilcoxon signed-rank test, given the non-normal data distribution. Then, patients’ baseline performances for the three sessions were compared using the non-parametric Friedman test, to check for the absence of inter-session learning effects. The results obtained in the different tasks before and immediately after tDCS were compared using the Wilcoxon signed-rank test for each stimulation modality: left-anodal, right-cathodal and sham, to assess stimulation effects. The total change in each task (post-stimulation – pre-stimulation) was compared between the three stimulation conditions using the Friedman test, to compare the effects of the different stimulation modalities.

With the aim of identifying potential individual factors influencing tDCS effects, we computed bivariate correlations (Spearman correlations) between changes in performance/RT in the different language tasks (post vs. pre-tDCS), and symptom duration and aphasia severity BDAE scores.

## 4 Results

### 4.1 MRI – voxel-based morphometry

**Fig. 1:**
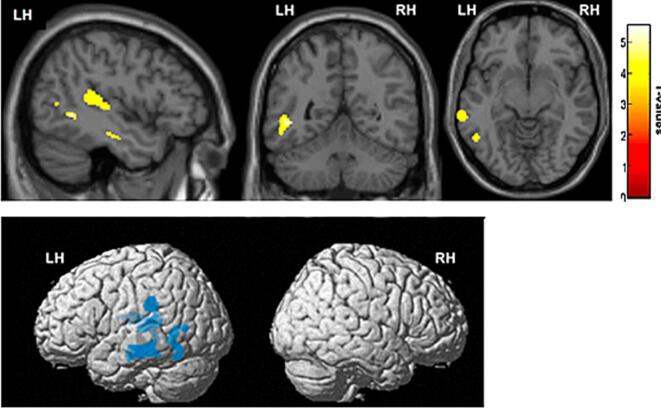
Voxel-based morphometry (VBM): Comparison between lv-PPA patients (N = 12) and healthy controls (N = 11) (p < 0.001, uncorrected). Top panel (from left to right): sagittal, coronal and axial slices showing atrophied voxel clusters. A central voxel cluster with the MNI coordinates [x = -45, y = -53, z = -2] (left posterior temporal cortex) survived with a statistical threshold of p < 0.05 corrected for multiple comparisons. The color bar represents t-values. Bottom panel: 3D reconstruction of the brain showing regions with gray matter thinning in the lv-PPA cohort (in blue), as compared to healthy controls. LH = left hemisphere, RH = right hemisphere.

Compared to controls, the patterns of significant gray matter atrophy in the lv-PPA cohort encompassed the left TPJ, including portions of the middle/posterior temporal cortex and the inferior parietal cortex (p < 0.001, uncorrected). A cluster of voxels with the MNI coordinates [x = −45, y = −53, z = −2] of the left posterior temporal cortex survived with a statistical threshold of p < 0.05, corrected for multiple comparisons (Fig. 1).

### 4.2 Computational model of current density distribution

**Fig. 2:**
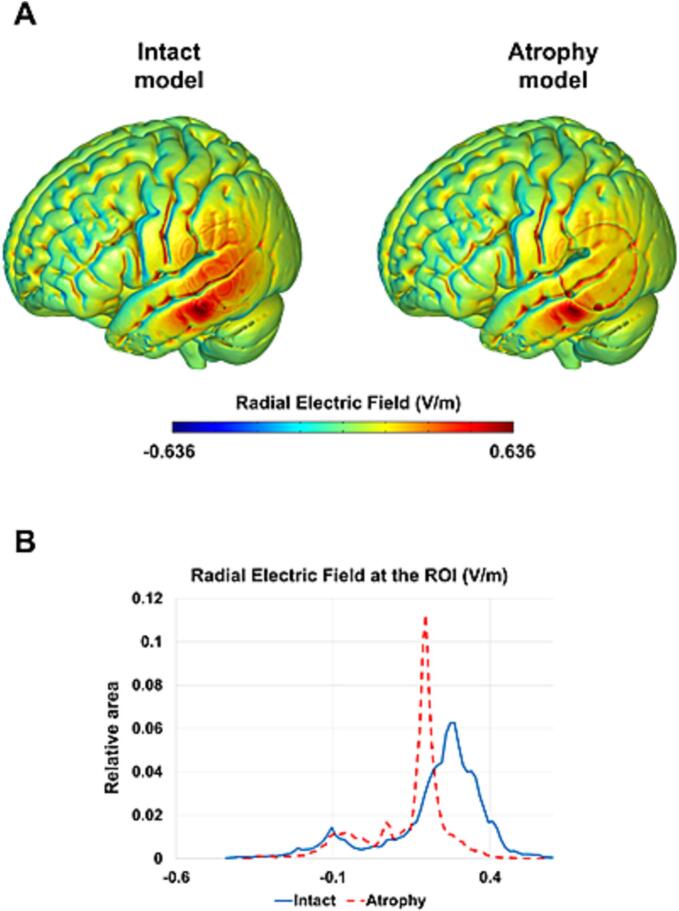
Results of computational finite element models simulating on a standard brain (ICBM-NY) the predicted radial electric field during left anodal tDCS, delivered with the electrode montage and parameters of the study targeting the TPJ (round 25 cm^2^ sponge electrodes [1.59 mA intensity, 0.06 mA/cm2 current density], with a contralateral supraorbital reference). (A) Radial electric field distribution (V/m) across the cortical surface in the model of an intact brain (left panel), and of a model implementing a 1.5 mm cortical thinning (atrophy) in a region of 5 cm of diameter within the TPJ (right panel). (B) Histogram of the predicted radial electric field in the region of interest (ROI, 5 cm diameter centered on the left TPJ, MNI coordinates [x = −64, y = −38, z = 6]), for the intact and the atrophy-implemented models (blue solid line and red dotted line, respectively).

Computer simulations predicted that both anodal and cathodal tDCS deliver current to the lateral aspects of the tDCS-targeted TPJ including posterior and middle temporal cortices and inferior parietal cortices. The peak of electrical field strength was located in the middle temporal gyrus for both models implementing ‘intact’ and ‘atrophied’ TPJ cortices. The model implementing atrophy of the left and right TPJ predicted a decrease in radial electric field compared to the ‘intact’ model (Fig. 2). Model simulations predicted that both active tDCS stimulation strategies (left-anodal, right-cathodal) differentially modulate activity of their respective TPJ targets. Directional current flow indicated opposite modulatory effects: left-anodal stimulation yielded enhancements of activity across the left TPJ and adjacent areas (current flow radially inward) and right-cathodal tDCS yielded relative decreases of activity across the right TPJ (current flow radially outward). The two tDCS modalities generated interhemispheric imbalance, with higher activity levels in left than right TPJ regions.

### 4.3 Language tasks at baseline

**Fig. 3:**
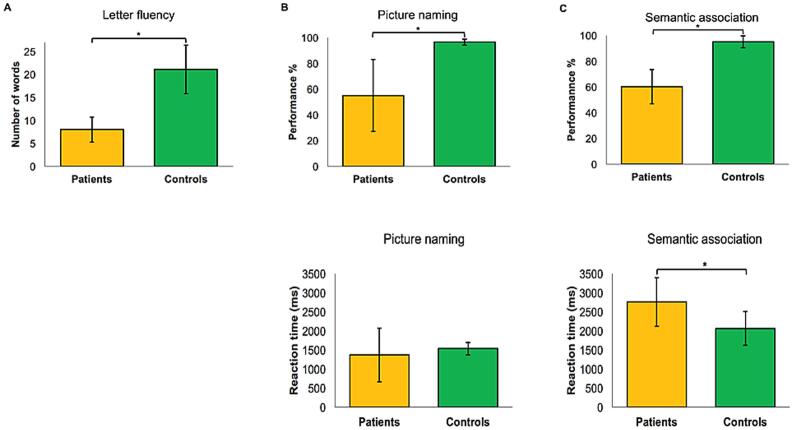
Pre-stimulation baseline performance (top panel) and reaction times (RT) (bottom panel) of lv-PPA patients and healthy controls (mean values ± standard deviations) in the three language tasks: (A) letter fluency task (LFT), (B) picture naming task (PNT), (C) semantic association task (SAT). Performance accuracy in patients was poorer than in controls in the three tasks (all p-values < 0.001). RT in patients were slower than in controls in the semantic association task (p = 0.003) but not in picture naming task (p > 0.05).

Patients showed significantly lower performance in the three language tasks as compared to healthy controls (all p-values < 0.001). Regarding RT, patients were slower than controls in the semantic association task (Z = −2.74, p = 0.003), but there was no difference in the PNT (p > 0.05). Results are shown in Fig. 3.

### 4.4 tDCS effects

Baseline performance measured during the pre-stimulation test sessions was similar for the three stimulation sessions (LFT: χ^2^(2) = 2.205, p = 0.332; PNT (performance-accuracy): χ^2^(2) = 1.721, p = 0.423; PNT (RT): χ^2^(2) = 2, p = 0.368; SAT (performance-accuracy): χ^2^(2) = 3.244, p = 0.197; SAT (RT): χ^2^(2) = 0.383, p = 0.826).

**Fig. 4:**
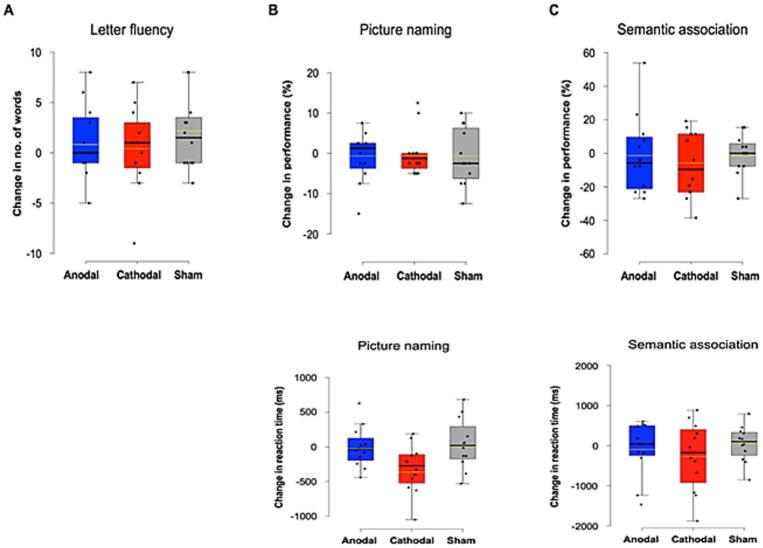
Changes between pre- and post-stimulation for performance (top panel) and reaction times (RT) (bottom panel) in the three language tasks: (A) letter fluency task (LFT), (B) picture naming task (PNT), (C) semantic association task (SAT). No significant performance improvement was found for any task (all p-values > 0.05). In the box-and-whisker plots, the boundary of the box closest to zero indicates the 25th percentile, a black line within the box marks the median, a yellow line within the box marks the mean, and the boundary of the box farthest from zero indicates the 75th percentile. Whiskers above and below the box indicate maximum and minimum values falling no more or no less than 1.5 times the length of the box. Points above and below the whiskers indicate outliers.

**Fig. 5:**
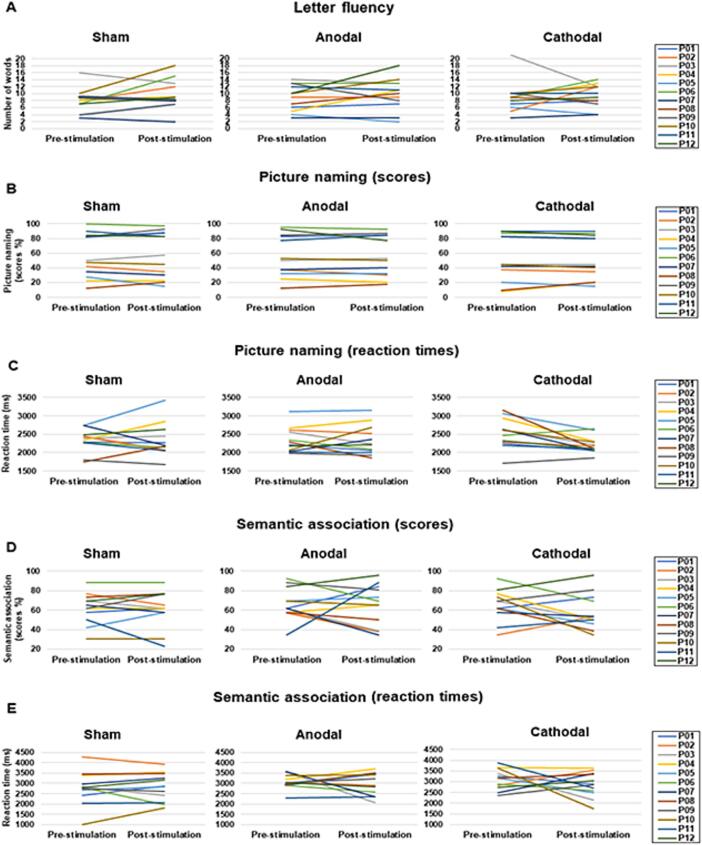
Individual results of the 12 lv-PPA patients at baseline (pre-tDCS) and post-tDCS for each task and each stimulation modality. A: letter fluency task (performance), B and C: picture naming task (performance and RT, respectively), D and E: semantic association task (performance and RT, respectively).

Analyses did not reveal statistically significant differences in post minus pre-tDCS performance or RT across the 3 stimulation modalities for any of the three experimental tasks (LFT: χ^2^(2) = 0.182, p = 0.913; PNT (performance-accuracy): χ^2^(2) = 0.044, p = 0.978; PNT (RT): χ^2^(2) = 4.5, p = 0.105; SAT (performance-accuracy): χ^2^(2) = 1.149, p = 0.563; SAT (RT): χ^2^(2) = 0.809, p = 0.668). Likewise, comparing separately post minus pre-tDCS performance for each stimulation modality did not show any significant effect of tDCS, whatever the stimulation modality, in any of the language tasks, regarding performance accuracy or RT (all p-values > 0.001) (Fig. 4). Detailed individual results for the comparison pre/post tDCS performance and RT for each patient, each task, and each stimulation modality are illustrated in Fig. 5.

### 4.5 Relationships between aphasia severity & disease duration and tDCS effects

Correlation analyses were performed given the variability of tDCS effects across patients. They revealed a significant positive association between changes in RT in the PNT (after cathodal stimulation) and aphasia severity scores (ρ = 0.659, p = 0.027), with faster post-tDCS than pre-tDCS RT being associated with lower aphasia severity scores. There was also a significant negative association between changes in performance in the SAT (after cathodal stimulation) and number of years of disease duration (ρ = −0.61, p = 0.035), with higher post-tDCS than pre-tDCS accuracy scores being associated with shorter disease durations. No other significant associations were found, and the above-mentioned results were not significant after correcting for multiple comparisons.

## 5 Discussion

To check for potential NIBS efficiency in PPA we used tDCS to improve language in a cohort of 12 clinically and imaging-characterized lv-PPA patients, adopting a double-blind sham-controlled approach, while applying a counter-balanced crossover design. We used two strategies: anodal tDCS over the left TPJ to directly boost language-related activity, and cathodal tDCS over the right TPJ to suppress its interhemispheric inhibitory effect on the left TPJ. Baseline results showed that patients had lower performance than controls in the three language tasks. Compared to sham neither left-anodal nor right-cathodal tDCS induced language modulations. Computational finite element models showed significant impact of left-anodal and right-cathodal tDCS on the TPJ (peak of current-field strength in the middle temporal gyrus), but they also revealed lower radial field strength when TPJ atrophy was implemented in the model. Correlation analyses with individual data indicated greater tDCS effects for picture-naming and semantic matching after cathodal stimulation for patients with shorter disease durations and lesser aphasia severity scores.

Beyond tDCS issues, our results showing low performance in the letter fluency task (lexical access mechanisms), the picture naming task (lexical/semantic access), and in the semantic association task (semantics), are consistent with previous studies (Gorno-Tempini et al., 2004, Teichmann et al., 2013) while further characterizing language deficits in lv-PPA, especially regarding semantic disorders. First, they reflect word-finding difficulties, which according to previous findings in lv-PPA, are related to impairment of lexical access/representations (Migliaccio et al., 2016, Sanches et al., 2018). Second, they suggest that such word-finding difficulties, after approximately 4 years of symptom evolution, also result from semantic impairments, thus supporting prior studies indicating semantic deficits in relatively early stages of lv-PPA (Rogalski et al., 2008, Teichmann et al., 2013, Migliaccio et al., 2016, Sanches et al., 2018). The existence of lexical/semantic deficits are consistent with imaging findings indicating that damage in lv-PPA is primarily located in lexical-related posterior temporal cortices (Gorno-Tempini et al., 2004, Teichmann et al., 2013, Routier et al., 2018), which expands, after 2–4 years of evolution, to semantic-related middle/anterior temporal regions (Rogalski et al., 2011, Wicklund et al., 2014, Routier et al., 2018, Leyton et al., 2013).

### 5.1 Factors influencing tDCS effects

We used the TPJ target since this region is primarily affected in lv-PPA, because it plays a key role in lexical processes, and because it is connected to semantic-related anterior temporal regions (Sundqvist et al., 2020). Furthermore, previous studies have indicated that NIBS over the TPJ improves performance in tasks involving lexical/semantic processing such as picture naming (Fiori et al., 2011, Roncero et al., 2017, Roncero et al., 2019). Despite this rationale, we did not find any tDCS effects. Hence, the crucial issue is to analyze the contrast between our ‘null effects’ and the positive effects reported by several prior tDCS studies in lv-PPA. A first general point is that methodologically rigorous studies providing evidence for ‘null effects’ are essential for counterbalancing potential publication-selection biases towards studies reporting positive effects. In other words, it might be that exhaustive communication of studies using NIBS in lv-PPA, or in other PPA variants, would have shown that a not negligible proportion of them might not have evidenced beneficial tDCS effects. An underlying question is why some studies do, and others do/might not report such effects. Providing answers to this issue is crucial for i) identifying parameters playing key roles in tDCS-induced language modulation, and ii) improving strategies for potential therapeutic tDCS use in lv-PPA, and more generally in PPA. Our investigation, in association with previous findings, reveals several of these parameters.

A first parameter is the stimulation site. Most prior NIBS studies in lv-PPA reporting beneficial outcomes have targeted prefrontal regions (Ficek et al., 2018, Tsapkini et al., 2014, Tsapkini et al., 2018), which are not part of the brain-language system. Nonetheless, they are involved in executive functioning that, among various cognitive dimensions, subtends access mechanisms to the linguistic system (Badre, 2008). These prefrontal regions are less atrophied in lv-PPA than the TPJ. A target choice of less atrophied regions is coherent with investigations indicating that atrophy impacts on tDCS current magnitude and current distribution over the cortex (Opitz et al., 2015). Our computational modeling results corroborate this issue showing a decrease in radial electric field strength in the model implementing TPJ atrophy. These findings encourage to re-think the rationale for brain targets, and to consider less atrophied but language-related target regions that might constitute the best gateways into the brain-language system. For example, the anterior temporal lobes (ATL), involved in verbal semantics, might be a more operational target given that the ATL of the dominant hemisphere is connected to lexical-related posterior temporal TPJ regions, which are atrophied in lv-PPA (Gorno-Tempini et al., 2004; 2011; Routier et al., 2018). The use of such less atrophied target regions is also supported by a prior study of our group, showing beneficial tDCS effects in semantic PPA especially when targeting the less atrophied right ATL with cathodal tDCS (Teichmann et al., 2016). A directly related parameter for tDCS efficiency is clinical aphasia severity, which increases with atrophy progression/severity. Hence, patients in early disease stages with less severe language impairments, related to lesser atrophy of the target region, might be the most appropriate candidates for tDCS. This clinical criterion appears to be corroborated by the results of our correlation analyses (not corrected for multiple comparisons) indicating that lv-PPA patients with less severe aphasia and shorter disease durations show more important tDCS effects than patients in later disease stages with more severe aphasia. Such correlation results more generally reflect the variability of tDCS effects across patients, which is further illustrated by individual findings indicating post-tDCS improvement in language tasks for several of our patients. Hence, ‘null results’ at the group level do not infer absence of tDCS efficiency as such, but they indicate the necessity to parametrize patient selection for tDCS along several dimensions including aphasia/atrophy severity and disease duration.

Second, studies reporting positive tDCS effects in lv-PPA showed effects only after combining NIBS with language training (Tsapkini et al., 2014, Tsapkini et al., 2018). This might reflect that modulatory effects of NIBS are determined by ongoing activity patterns within the target region at the time of stimulation, known as ‘activity state dependent effects’ (Silvanto et al., 2008). One critical parameter might therefore be that positive tDCS effects depend on initial priming of neural activity in the target region, and the related cognitive system, which could be obtained by online language tasks or iterative/intense langue training. Hence, researchers should consider the combination of NIBS with language training or online tasks, which specifically impact on those components of the brain-language system that constitute the intended tDCS targets (Cotelli et al., 2020).

A third parameter is the stimulation regime (single sessions vs. multi-day NIBS) given that only iterative tDCS appears to result in cumulative, lasting, and potentially therapeutic effects, via the promotion of brain plasticity (Monte-Silva et al., 2013). Accordingly, most tDCS studies in lv-PPA reporting beneficial effects have applied multi-day regimes (Gervits et al., 2016, Ficek et al., 2018, Tsapkini et al., 2014, Tsapkini et al., 2018). However, although iterative tDCS sessions might generate lasting effects, significant offline language modulation has also been reported in proof-of-concept studies with PPA using single NIBS sessions within cross-over designs as in our investigation (Cotelli et al., 2012, Teichmann et al., 2016).

Another factor that might explain the discrepancy between our ‘null results’ and prior studies having reported positive tDCS effects in lv-PPA could be related to the number of explored patients given that different patient sample sizes can yield different results, and that larger sample sizes provide more statistically reliable findings. However, sample sizes in previous studies were similar and in most of them even smaller than in our investigation. The largest reported sample size included, like in our study, 12 lv-PPA patients (Tsapkini et al., 2018), and positive tDCS effects were observed specifically on spelling performances when tDCS over prefrontal regions was coupled with language training. Other studies with slightly larger lv-PPA sample sizes combined different PPA variants in statistical analyses, without providing detailed results regarding lv-PPA patients (e.g., Wang et al., 2023).

Finally, a critical factor impacting on outcomes is the choice of language markers assessing tDCS effects. One limitation of the present investigation was to tap only lexical-semantic dimensions, without assessing sentence repetition, which is another core feature of lv-PPA (Gorno-Tempini et al., 2011). More generally, language markers should assess the main features of each PPA variant, and additional ‘ecological’ questionnaires should be used to evaluate potential meaningful language/communication impacts of tDCS in everyday life.

## 6 Conclusion

Our study mitigates the message of beneficial language effects of tDCS in lv-PPA. However, and more importantly, it opens avenues for future research, which should investigate and identify the critical parameters influencing tDCS efficiency to delineate strategies that could lead to future therapeutic use of tDCS in lv-PPA and possibly in other PPA variants. Our findings mainly call attention to two issues: (1) The choice of brain targets for tDCS, favoring less atrophied language-related regions, as supported by our results of computational modelling implementing cortical atrophy. Such tDCS targets might constitute appropriate gateways into the brain-language network ensuring efficient current magnitude and distribution, and significant language effects. (2) The selection of candidates for tDCS, probably favoring PPA patients having slight atrophy and aphasia severity, as suggested by the combined results of our computational modelling and uncorrected correlation results with individual data. Future studies should further explore these issues, which might lead to customized tDCS use, and open the way towards robust proof-of-concept, which is necessary for subsequent therapy validation investigations in large patient cohorts using multi-day tDCS regimes.

## CRediT authorship contribution statement

**Marc Teichmann:** Writing – original draft, Visualization, Validation, Supervision, Resources, Project administration, Methodology, Investigation, Funding acquisition, Conceptualization. **Clara Sanches:** Writing – review & editing, Visualization, Software, Methodology, Funding acquisition, Formal analysis. **Angelina Bourbon:** Writing – review & editing, Data curation. **Dennis Q. Truong:** Writing – review & editing, Software, Methodology, Investigation, Formal analysis. **Marom Bikson:** Writing – review & editing, Software, Methodology, Formal analysis, Conceptualization. **Antoni Valero-Cabré:** Writing – review & editing, Visualization, Validation, Supervision, Software, Resources, Project administration, Methodology, Investigation, Funding acquisition, Conceptualization.

## Funding

Clara Sanches was funded by PhD funds from the ‘Fondation Recherche Alzheimer’ (FRA). The activities of Antoni Valero-Cabré’s research team were supported by research grants ‘IHU-A-ICM-Translationnel’, ‘Agence Nationale de la Recherche’ (ANR), ‘Projet OSCILOSCOPUS’, and the ‘PHRC NEGLECT’.

## Declaration of Competing Interest

The authors declare that they have no known competing financial interests or personal relationships that could have appeared to influence the work reported in this paper.

## Data Availability

Data will be made available on request.

## References

[b0005] Badre D. (2008). Cognitive control, hierarchy, and the rostro–caudal organization of the frontal lobes. Trends Cogn. Sci..

[b0010] Bonin P., Peereman R., Malardier N., Méot A., Chalard M. (2003). A new set of 299 pictures for psycholinguistic studies: French norms for name agreement, image agreement, conceptual familiarity, visual complexity, image variability, age of acquisition, and naming latencies. Behav. Res. Methods. Instrum. Comput..

[b0015] Brunoni A.R., Amadera J., Berbel B., Volz M.S., Rizzerio B.G., Fregni F.A. (2011). Systematic review on reporting and assessment of adverse effects associated with transcranial direct current stimulation. Int. J. Neuropsychopharmacol..

[b0020] Cardebat D., Doyon B., Puel M., Goulet P., Joanette Y. (1990). Literal and category word fluency in normal subjects. Performance and dynamics of word production as a function of gender, age and educational level. Acta Neurol. Belg..

[b0025] Cotelli M., Manenti R., Alberici A., Brambilla M., Cosseddu M., Zanetti O., Miozzo A., Padovani A., Miniussi C., Borroni B. (2012). Prefrontal cortex rTMS enhances action naming in progressive non-fluent aphasia. Eur. J. Neurol..

[b0030] Cotelli M., Manenti R., Petesi M., Brambilla M., Cosseddu M., Zanetti O., Miniussi C., Padovani A., Borroni B. (2014). Tre. J. Alzheimers. Dis..

[b0035] Cotelli M., Manenti R., Ferrari C., Gobbi E., Macis A., Cappa S.F. (2020). Effectiveness of language training and non-invasive brain stimulation on oral and written naming performance in Primary Progressive Aphasia: A meta-analysis and systematic review. Neurosci. Biobehav. Rev..

[b0040] Deloche G., Hannequin D. (1997).

[b0045] Dubois B., Slachevsky A., Litvan I., Pillon B. (2000). The FAB: a Frontal Assessment Battery at bedside. Neurology.

[b0050] Elder G., Taylor J.P. (2014). Transcranial magnetic stimulation and transcranial direct current stimulation: treatments for cognitive and neuropsychiatric symptoms in the neurodegenerative dementias?. Alzheimers. Res. Ther..

[b0055] Ferbert A., Priori A., Rothwell J.C., Day B.L., Colebatch J.G., Marsden C.D. (1992). Interhemispheric inhibition of the human motor cortex. J. Physiol..

[b0060] Ficek B.N., Wang Z., Zhao Y., Webster K.T., Desmond J.E., Hillis A.E., Frangakis C., Vasconcellos Faria A., Caffo B., Tsapkini K. (2018). The effect of tDCS on functional connectivity in primary progressive aphasia. Neuroimage Clin..

[b0065] Fiori V., Coccia M., Marinelli C.V., Vecchi V., Bonifazi S., Ceravolo M.G., Provinciali L., Tomaiuolo F., Marangolo P. (2011). Transcranial direct current stimulation improves word retrieval in healthy and nonfluent aphasic subjects. J. Cogn. Neurosci..

[b0070] Folstein M., Folstein S., McHugh P.R. (1975). Mini-Mental state: a practical method for grading the cognitive state of patients for the clinician. J. Psychiatr. Res..

[b0075] Gervits F., Ash S., Coslett H.B., Raskovsk K., Grossman M., Hamilton R. (2016). Transcranial direct current stimulation for the treatment of primary progressive aphasia: An open-label pilot study. Brain. Lang..

[b0080] Gorno-Tempini M.L., Hillis A.E., Weintraub S., Kertesz A., Mendez M., Cappa S.F., Ogar J.M., Rohrer J.D., Black S., Boeve B.F., Manes F., Dronkers N.F., Vandenberghe R., Rascovsky K., Patterson K., Miller B.L., Knopman D.S., Hodges J.R., Mesulam M.M., Grossman M. (2011). Classification of primary progressive aphasia and its variants. Neurology.

[b0085] Gorno-Tempini M.L., Dronkers N.F., Rankin K.P., Ogar J.M., Phengrasamy L., Rosen H.J., Johnson J.K., Weiner M.W., Miller B.L. (2004). Cognition and anatomy in three variants of primary progressive aphasia. Ann. Neurol..

[b0090] Hamilton R.H., Chrysikou E.G., Coslett B. (2011). Mechanismes of aphasia recovery after stroke and therole of noninvasive brain stimulation. Brain. Lang..

[b0095] Huang Y., Parra L.C., Haufe S. (2016). The New York head-A precise standardized volume conductor model for EEG source localization and tES targeting. NeuroImage.

[b0100] Leyton C.E., Hsieh S., Mioshi E., Hodges J.R. (2013). Cognitive decline in logopenic aphasia: more than losing words. Neurology.

[b0105] Mazaux J.M., Orgogozo J.M. (1982).

[b0110] Mesulam M.M., Rogalski E.J., Wieneke C., Hurley R.S., Geula C., Bigio E.H., Thompson C.K., Weintraub S. (2014). Primary progressive aphasia and the evolving neurology of the language network. Nat. Rev. Neurol..

[b0115] Migliaccio R., Boutet C., Valabregue R., Ferrieux S., Nogues M., Lehéricy S., Dormont D., Levy R., Dubois B., Teichmann M. (2016). The brain network of naming: A lesson from primary progressive aphasia. PloS One.

[b0120] Monte-Silva K., Kuo M.F., Hessenthaler S., Fresnoza S., Liebetanz D., Paulus W., Nitsche M.A. (2013). Induction of late LTP-like plasticity in the human motor cortex by repeated non-invasive brain stimulation. Brain. Stimul..

[b0125] Montgomery S.A., Asberg M.A. (1979). New depression scale designed to be sensitive to change. Br. J. Psychiatry..

[b0130] Monti A., Cogiamanian F., Marceglia S., Ferrucci R., Mameli F., Mrakic-Sposta S., Vergari M., Zago S., Priori A. (2008). Improved naming after transcranial direct current stimulation in aphasia. J. Neurol. Neurosurg. Psychiatry..

[b0135] New B., Pallier C., Ferrand L., Matos R. (2004). Une base de données lexicales du français contemporain sur internet: LEXIQUE 2. Année Psychol.

[b0140] Nitsche M.A., Paulus W. (2001). Sustained excitability elevations induced by transcranial DC motor cortex stimulation in humans. Neurology.

[b0145] Opitz A., Paulus W., Will S., Antunes A., Thielscher A. (2015). Determinants of the electric field during transcranial direct current stimulation. NeuroImage.

[b0150] Patel N., Peterson K.A., Ingram R.U., Storey I., Cappa S.F., Catricala E., Halai A., Patterson K.E., Lambon Ralph M.A., Rowe J.B., Garrard P. (2021). A 'Mini Linguistic State Examination' to classify primary progressive aphasia. Brain. Commun..

[b0155] Rogalski E., Rademaker A., Mesulam M., Weintraub S. (2008). Covert processing of words and pictures in nonsemantic variants of Primary Progressive Aphasia. Alzheimer. Dis. Assoc. Disord..

[b0160] Rogalski E., Cobia D., Harrison T.M., Wieneke C., Weintraub S., Mesulam M.M. (2011). Progression of language decline and cortical atrophy in subtypes of primary progressive aphasia. Neurology.

[b0165] Roiser J.P., Wigton K.JM., Mendez M.A., Hon N., Friston K.J., Joyce E.M. (2013). Dysconnectivity in the frontoparietal attention network in schizophrenia. Front. Psychiatry..

[b0170] Roncero C., Kniefel H., Service E., Thiel A., Probst F., Chertkow H. (2017). Inferior parietal transcranial direct current stimulation with training improves cognition in anomic Alzheimer’s disease and frontotemporal dementia. Alzheimers Dementia (NY).

[b0175] Roncero C., Service E., De Caro M., Popov A., Thiel A., Probst S., Chertkow H. (2019). Maximizing the treatment benefit of tDCS in neurodegenerative anomia. Front. Neurosci..

[b0180] Routier A., Habert M.O., Bertrand A., Kas A., Sundqvist M., Mertz J., David P.M., Bertin H., Belliard S., Pasquier F., Bennys K., Martinaud O., Etcharry-Bouyx F., Moreaud O., Godefroy O., Pariente J., Puel M., Couratier P., Boutoleau-Bretonnière C., Laurent B., Migliaccio R., Dubois B., Colliot O., Teichmann M. (2018). Structural, microstructural, and metabolic alterations in primary progressive aphasia variants. Front. Neurol..

[b0185] Sanches C., Routier A., Colliot O., Teichmann M. (2018). The structure of the mental lexicon: What primary proressive aphasias reveal. Neuropsychologia.

[b0190] Sanches C., Stengel C., Godard J., Mertz J., Teichmann M., Migliaccio R., Valero-Cabré A. (2021). Past, present and future of non-invasive brain stimulation approaches to treat cognitive impairment in neurodegenerative diseases: time for a comprehensive critical systematic review. Front. Aging. Neurosci..

[b0195] Sanches C., Amzallag F., Dubois B., Lévy R., Truong D.Q., Bikson M., Teichmann M., Valero-Cabré A. (2022). Evaluation of the effect of transcranial direct current stimulation on language impairments in the behavioral variant of frontotemporal dementia. Brain. Commun..

[b0200] Silvanto J., Muggleton N., Walsh V. (2008). State-dependency in brain stimulation studies of perception and cognition. Trends. Cogn. Sci..

[b0205] Snodgrass J.G., Vanderwart M. (1980). A standardized set of 260 pictures: norms for name agreement, image agreement, familiarity, and visual complexity. J. Exp. Psychol. Hum. Learn..

[b0210] Sundqvist M., Routier A., Dubois B., Colliot O., Teichmann M. (2020). The white matter module-hub network of semantics revealed by semantic dementia. J. Cogn. Neurosci..

[b0215] Teichmann M., Kas A., Boutet C., Ferrieux S., Nogues M., Samri D., Rogan C., Dormont D., Dubois B., Migliaccio R. (2013). Deciphering logopenic primary progressive aphasia: a clinical, imaging and biomarker investigation. Brain.

[b0220] Teichmann M., Lesoil C., Godard J., Vernet M., Bertrand A., Levy R., Dubois B., Lemoine L., Truong D.Q., Bikson M., Kas A., Valero-Cabré A. (2016). Direct current stimulation over the anterior temporal areas boosts semantic processing in primary progressive aphasia. Ann. Neurol..

[b0225] Torres J., Drebing D., Hamilton R. (2013). TMS and tDCS in post-stroke aphasia: Integrating novel treatment approaches with mechanisms of plasticity. Restor. Neurol. Neurosci..

[b0230] Tsapkini K., Frangakis C., Gomez Y., Davis C., Hillis A.E. (2014). Augmentation of spelling therapy with transcranial direct current stimulation in primary progressive aphasia: Preliminary results and challenges. Aphasiology.

[b0235] Tsapkini K., Webster K.T., Ficek B.N., Desmond J.E., Onyike C.U., Rapp B., Frangakis C.E., Hillis A.E. (2018). Electrical brain stimulation in different variants of primary progressive aphasia: A randomized clinical trial. Alzheimers. Dementia. (NY).

[b0240] Valero-Cabré A., Sanches C., Godard J., Fracchia O., Dubois B., Levy R., Truong D.Q., Bikson M., Teichmann M. (2019). Language boosting by transcranial stimulation in Progressive Supranuclear Palsy. Neurology.

[b0245] Wang Z., Ficek B.N., Webster K.T., Herrmann O., Frangakis C.E., Desmond J.E., Onyike C.U., Caffo B., Hillis A.E., Tsapkini K. (2023). Specificity in generalization effects of transcranial direct current stimulation over the left frontal gyrus in primary progressive aphasia. Neuromudulation.

[b0250] Wicklund M.R., Duffy J.R., Strand E.A., Machulda M.M., Whitwell J.L., Josephs K.A. (2014). Quantitative application of the primary progressive aphasia consensus criteria. Neurology.

